# T-psi-C: user friendly database of tRNA sequences and structures

**DOI:** 10.1093/nar/gkz922

**Published:** 2019-10-18

**Authors:** Marcin Piotr Sajek, Tomasz Woźniak, Mathias Sprinzl, Jadwiga Jaruzelska, Jan Barciszewski

**Affiliations:** 1 Institute of Human Genetics, Polish Academy of Sciences, Strzeszynska 32, 60-479, Poznan, Poland; 2 Bayreuth University, Bayreuth, Germany; 3 Institute of Bioorganic Chemistry of the Polish Academy of Sciences, Noskowskiego 12, 61-704 Poznan, Poland; 4 NanoBioMedical Centre of the Adam Mickiewicz University, Umultowska 85, 61-614 Poznan, Poland

## Abstract

tRNAs have been widely studied for their role as genetic code decoders in the ribosome during translation, but have recently received new attention due to the discovery of novel roles beyond decoding, often in connection with human diseases. Yet, existing tRNA databases have not been updated for more than a decade, so they do not contain this new functional information and have not kept pace with the rate of discovery in this field. Therefore, a regularly updated database that contains information about newly discovered characteristics of tRNA molecules and can be regularly updated is strongly needed. Here, we report the creation of the T-psi-C database (http://tpsic.igcz.poznan.pl), an up-to-date collection of tRNA sequences that contains data obtained from high-throughput tRNA sequencing, e.g. all isoacceptors and isodecoders for human HEK293 cells. This database also contains 3D tRNA structures obtained from Protein Data Bank and generated using homology modeling. The T-psi-C database can be continuously updated by any member of the scientific community, and contains its own application programming interface (API), which allows users to retrieve or upload data in JSON format. Altogether, T-psi-C is user-friendly, easy to develop and an up-to-date source of knowledge about tRNAs.

## INTRODUCTION

The role of tRNAs as genetic code decoders has been widely studied since the mid-1950s, and is well understood (for a review, see (1)). Currently, the tRNA research field is experiencing a renaissance, and much of this interest has focused on the tissue-specific regulation of translation decoding efficiency, due to expression level changes and/or nucleoside modification, which are often connected with human disease. For example, changes in tRNA isoacceptor (tRNA with distinct anticodon sequences loaded with the same amino acid) and/or isodecoder (the same anticodon sequence, but differences in tRNA body) expression levels may dramatically influence cell physiology, and in breast cancer cells, high expression of tRNAGlu/UUC and tRNAArg/CCG isoacceptors causes a shift from normal protein expression towards an expression profile that promotes metastasis (2). Similarly, downregulation of one of the five tRNAArg/UCU isodecoders, which is important for neuronal homeostasis, causes neurodegeneration (3). Additionally, several human diseases are associated with a lack of modified nucleosides that are normally present in particular tRNAs (for a review, see (4)). Finally, over 200 mutations in human mitochondrial genes encoding tRNAs have been identified as being associated with various human pathologies, such as myopathy, hearing loss, encephalopathy or gastrointestinal dysmotility (for reviews, see (5,6)). Therefore, there is a tremendous amount of new information that incorporates diverse aspects of tRNAs, such as sequences, structures, nucleoside modifications and isoacceptor and isodecoder expression levels, that is currently available in the scientific literature. Yet, this new information has not, been compiled into a single, up-to-date source or database.

Currently, two main databases are used by tRNA researchers: tRNAdb (7) and GtRNAdb (8,9). Starting from late 1970s a concerted effort was made to collect all known tRNA sequences in one place and to analyze structural and functional relationships between them (10–12). The result of this effort was the ‘Compilation of tRNA sequences’ database that was systematically updated by the creators, and made available as downloadable MS-Excel spreadsheets (13). In 2009, the database was transferred to a MySQL webpage (http://trnadb.bioinf.uni-leipzig.de) (7), updated to include several improvements (such as a user-friendly interface) and renamed tRNAdb. However, no new tRNA sequences have been added to this database since 2007, which means that several tRNA sequences obtained after this date, including all derived from high-throughput RNA-Seq methods are missed in tRNAdb. Another tRNA database—genomic tRNA Database (GtRNAdb, recently in version 2.0) (http://gtrnadb.ucsc.edu/) (8,9) contains tRNA predictions based on complete or nearly complete genomes. Nonetheless, the annotation process of this database is automated, and new entries are not checked for agreement with the published literature. Prediction is made at the genomic level. For the organisms with unknown tRNA expression profiles GtRNAdb data require further experimental validation to confirm expression of particular tRNA.

Importantly, in addition to their other limitations, neither of these databases contains structural information about tRNA molecules. At present, this information can only be retrieved from general structural databases, primarily the Protein Data Bank (PDB), and while many solved tRNA structures are available in PDB, the majority were obtained in complex with one or multiple proteins. This makes extracting tRNA structures from PDB files challenging because it requires specialized knowledge about PDB file structure, including programming skills that may be non-intuitive and problematic for an inexperienced user.

Here, we present the T-psi-C database (http://tpsic.igcz.poznan.pl), which contains sequences of tRNA molecules from organisms and viruses obtained by several methods of tRNA sequencing. It also includes their 2D and in most cases 3D structures as well as information about modified nucleosides. T-psi-C database offers three major advantages over previous tRNA databases: (i) an updated set of tRNA sequences, including data from high-throughput techniques; (ii) 3D structures of tRNA molecules retrieved from PDB or 3D structural models generated by homology modeling; (iii) the capacity for continuous updating with newly obtained tRNA sequences and structures by any member of the scientific community. We expect that this database will serve as a valuable resource for the scientific community, including molecular biologists, crystallographers, geneticists, chemists, computational biologists and others as the pace of tRNA research continues to increase, and we uncover new roles for these multifunctional molecules.

## DATABASE CONTENT

T-psi-C is manually curated collection of tRNA sequences and structures. Importantly, it contains sequences obtained from RNA sequencing and not from computational prediction based on genomic sequences. tRNA sequences present in the tRNAdb, except for computationally predicted ‘tRNA genes’ (7), were imported into T-psi-C. Sequences were corrected, if necessary, based on recently published literature, e.g., several modified nucleosides were added in the place of an unknown modification, 2D structures of selenocysteine tRNAs were re-aligned according to the recent structural data. A literature search was performed to add tRNA sequences published in the last decade as well as sequences that were published earlier, but were not present in tRNAdb. Finally T-psi-C database contain 1030 tRNA sequences.

The recent development of high-throughput RNA sequencing, including hydro-tRNAseq technology, has allowed the sequences of the entire tRNA transcriptomes of fission yeast (14) and human HEK293 cells (15) to be obtained. Therefore, the T-psi-C database contains 251 sequences based on hydro-tRNAseq. In the near future, we plan to add data from high-throughput methods other than hydro-tRNAseq, such as ARM-Seq (16), DM-tRNA-seq (17), YAMAT-seq (18) and mim-tRNAseq (19).

Structural data retrieved from Protein Data Bank and generated by homology modeling were also incorporated into T-psi-C. First, PDB was searched to obtain all tRNA structures. In a second step, tRNA 3D structures were extracted from tRNA-protein complexes and tRNA sequences were retrieved from 3D structures using RNApdbee (20,21). Sequences retrieved from structural data were compared with those present in T-psi-C database. If the sequence was present in the T-psi-C database, 3D structural data were added to the sequence record. If a particular sequence was absent (46 cases), it was retrieved from the 3D structure and added to T-psi-C along with the structural data. If 3D structures were not available, structural models for majority of sequences were generated using ModeRNA (22,23), followed in most cases by MMTK (dirac.cnrs-orleans.fr/MMTK.html) for energy minimization, and the resulting structures were entered into T-psi-C. Thus, for 899 from 1030 tRNA molecules present in T-psi-C, the sequence as well as the 3D structure can be retrieved.

A comparison between the content of T-psi-C and existing tRNA databases is shown in Table 1.

**Table 1.** Comparison between the data content in existing tRNA databases: T-psi-C, tRNAdb (7) and GtRNAdb 2.0* (9)

Feature	T-psi-C	tRNAdb	GtRNAdb 2.0
Total number of tRNA sequences, including:	1030	623	–
mitochondrial tRNAs	122	111	–
plastid tRNAs	38	38	–
viral tRNAs	17	17	–
cytoplasmic	853	457	–
tRNAs obtained from hydro-tRNAseq	251	–	–
tRNAs with structural data from crystal structure	54	–	–
tRNAs obtained only from PDB	46	–	–
tRNAs predicted from genomic sequences	–	12099	392089^a^

^a^Retrieved 18 July 2019.

Upper part of the table compare total number of tRNA molecules from distinct cellular compartments/viruses. Lower part show distinct data sources used in compared databases.

## DATABASE ORGANIZATION AND FUNCTIONALITIES

The core of the T-psi-C database was created using the MySQL, Django framework with the Django Rest Framework and Python programing language.

A user-friendly search interface was designed to allow easy and rapid retrieval of interesting data. In addition to ‘tRNA ID’, ‘tRNA type’, ‘Organism name’, ‘coding AA’ and ‘Anticodon sequence’, the database can also be searched using the sequence or 2D structure as a query.

For sequence searching, two different approaches were implemented: (i) the very rapid and efficient Elasticsearch (https://www.elastic.co/) approach using the n-gram model and (ii) the commonly used BLAST approach (BLAST+ version 2.7.1) (24). In the case of the n-gram model, many more results are displayed; however, the score generated by Elasticsearch enables easy selection of significant results, which are displayed as a list of individual records. Moreover, Elasticsearch-based implementation allows a direct search for modified sequences, which is not supported by BLAST. On the other hand results of the BLAST search represent only significant hits together with sequence alignment. The search results are displayed as a list of individual records.

For all records, the ID system from tRNAdb 2009 was retained, with the prefixes ‘tdbR’ and ‘tdbPDB’ added for records obtained from sequencing and structural data, respectively.

## THE DATABASE RECORD

Each individual database record represents a unique tRNA molecule from a particular species/organ/cell line. Each record contains information about the organism, amino acid specificity, tRNA cellular origin (cytoplasmic, mitochondrial, plastid or viral) and anticodon sequence. Each record also contains a full tRNA sequence with unmodified nucleosides—a so-called ‘flat sequence’, a secondary structure in dot-bracket format and sequence and secondary structure pre-aligned to the universal tRNA template. For some records, additional information is given in the ‘Comments’ field. Bibliographic information for the publication from which a sequence/structure is derived is given in the ‘Publications’ field, with a links to source publications in PubMed.

The 2D structure is depicted in the cloverleaf form, generated using custom-made JavaScript code, and displays nucleotides at sites specified by XML generated by TRAVeLer (25). Clicking on a modified nucleoside in the 2D structure displays a MODOMICS (26) cross-reference containing more details about that particular modification. Due to the large number of modified nucleosides, they are identified within the 2D sequence using the MODOMICS abbreviation system (http://modomics.genesilico.pl/modifications/), which is an extension of the ASCII-based system used in tRNAdb.

Folding free energy was calculated for each tRNA secondary structure using ViennaRNA (RNAlib-2.4.14) (https://www.tbi.univie.ac.at/RNA/ViennaRNA/doc/html/wrappers.html).

The 3D structure is depicted using a Jmol applet (27), allowing for zooming and rotating. The 3D structures can be downloaded in PDB format. For structural data retrieved from PDB, PDB IDs and cross-references to the PDB database are given.

**Figure 1.**
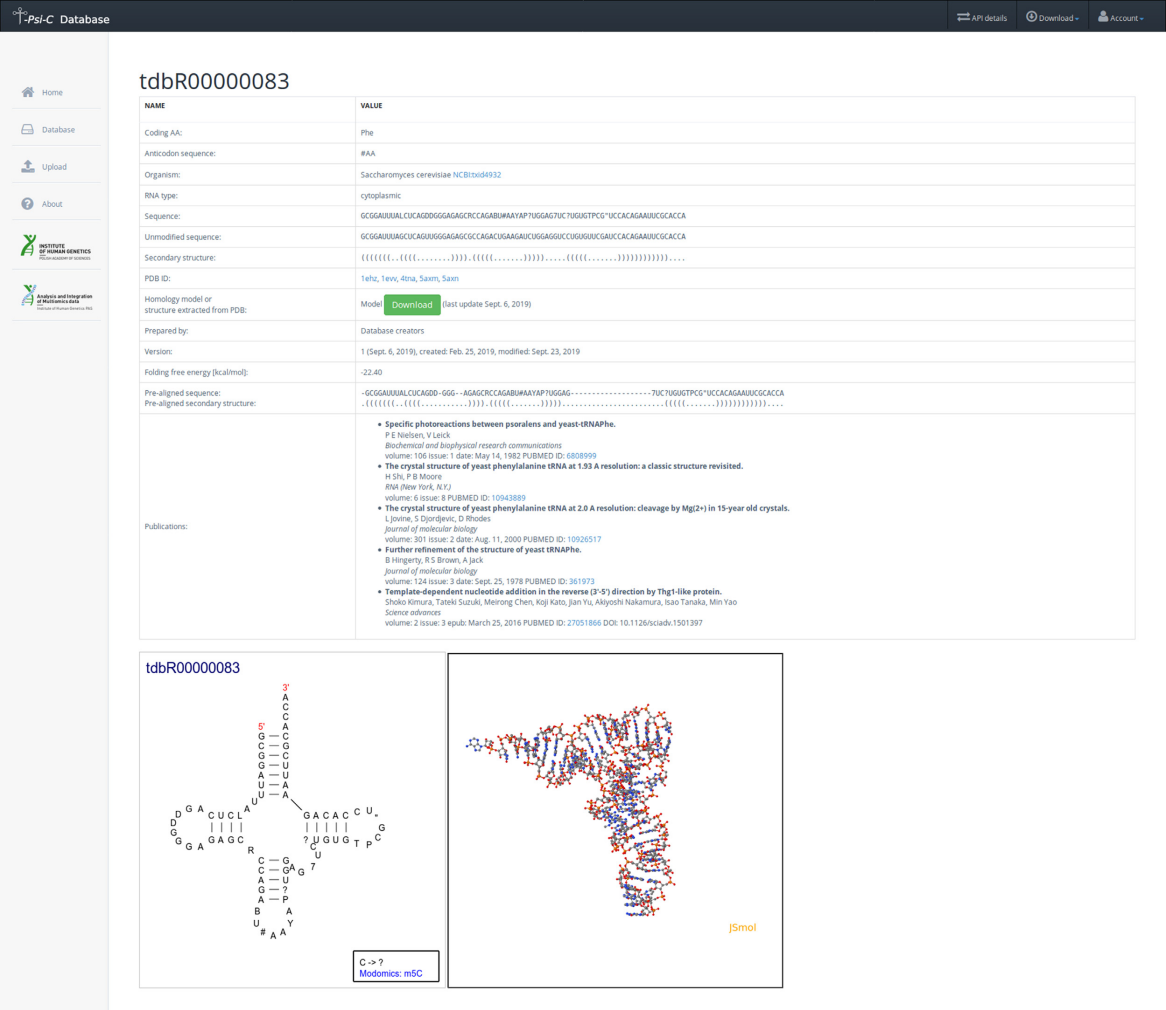
Representative record of the T-psi-C database showing model molecule – phenylalanine tRNA GmAA from *Saccharomyces cerevisiae*. Molecule in cloverleaf presentation and 3D crystal structure are visible in the bottom part of the picture.

Representative record is showed in Figure 1.

## ADDING NEW RECORDS AND DATA RETRIEVAL

Users can add new entries to the T-psi-C via the ‘add new entry’ option once they have registered for an account. The registered user then completes the form, including the PubMed ID of the publication containing information about the tRNA sequence/structure, for verification purposes. There is requirement that publication contains information how the published sequence was obtained, including experimental details and computational pipeline, if necessary. Detailed guidelines for adding new records can be found at http://tpsic.igcz.poznan.pl/info/add-entry/. Upon positive verification ensuring that a particular sequence does not exist in the database and if the user provided data are in agreement with data in publication, the record will be publicly displayed. If structural data is not provided by the user, a 3D structure will be generated automatically by homology modeling. The T-psi-C database contains its own application programming interface (API) that allows registered users to retrieve or upload data in JSON format. The use of this API is highly recommended in the case of future integration with other tools.

## DISCUSSION AND CONCLUSIONS

The T-psi-C database assembles several layers of information about tRNAs. This information includes sequences, secondary and tertiary structures and modifications. T-psi-C provides user-friendly interface with sequence and 2D structure search and rapid retrieval and download of interesting data. However, for the database to become as complete a source of tRNA knowledge as possible, it requires regular updates. The architecture of T-psi-C allows for easily adding new information.

The T-psi-C database, in contrary to tRNAdb and GtRNAdb, contains only sequences obtained from RNA sequencing, which is a way to confirm expression of particular tRNA molecule present in the database in particular tissue or organism. tRNA sequences obtained from *in silico* prediction of sequenced genomes were omitted. T-psi-C contains a higher number of tRNA sequences than tRNAdb ‘tRNA sequences’ section (1030 versus 623, for details see Table 1), including most recently published ones. Some of these new sequences were generated using high-throughput transcriptomic methods. We expect that the number of sequences in the T-psi-C database provided by high-throughput techniques will increase rapidly. We also believe that fast and efficient sequencing methods of tRNA molecules together with modified nucleoside identification will be possible in the near future, e.g. by using nanopore sequencing technology, which has been successfully used to identify some modified nucleosides in *Escherichia coli* 16S rRNA (28).

In the T-psi-C database, tRNA sequence is linked with 3D structure obtained from PDB or generated by homology modeling. Such data were absent in previous tRNA databases. At the present time, 3D structures or models are available for 899 sequences. The remaining part represents tRNAs with non-canonical 3D structures (usually mitochondrial), which are difficult to modeling. We are currently working on programming solutions to overcome this problem and generate the missing models as well as improve some of existing ones.

A unique feature of the T-psi-C database in comparison to previous tRNA databases, is the possibility of continuous update by any member of scientific community. It will help to keep the database up-to-date, especially in the ‘omics’ technologies era, when large amount of data are generated in a very short time.

The T-psi-C database can be further developed by adding information about the level of tRNA expression in different tissues, of eukaryotic organisms, pathological tRNA mutations, sites of interactions with proteins, sequences of tRNA precursors, rendering it even more useful for the scientific community. Therefore we would appreciate it if users add such annotations in their contributed database records.

## DATA AVAILABILITY

The T-psi-C database is freely available at http://tpsic.igcz.poznan.pl. All suggestions, corrections, comments and new entries are welcome.
